# Patterns of antidepressant prescribing around pregnancy: a descriptive analysis using Clinical Practice Research Datalink GOLD

**DOI:** 10.3399/BJGP.2025.1093

**Published:** 2025-12-16

**Authors:** Florence Z Martin, Gemma C Sharp, Kayleigh E Easey, Paul Madley-Dowd, Liza Bowen, Victoria Nimmo-Smith, Aws Sadik, Jonathan L Richardson, Dheeraj Rai, Harriet Forbes

**Affiliations:** 1 MRC Integrative Epidemiology Unit, University of Bristol, Bristol, UK; 2 Population Health Sciences, Bristol Medical School, University of Bristol, Bristol, UK; 3 Centre for Academic Mental Health, University of Bristol, Bristol, UK; 4 School of Psychology, University of Exeter, Exeter, UK; 5 School of Psychological Sciences, University of Bristol, Bristol, UK; 6 NIHR Bristol Biomedical Research Centre, University Hospitals Bristol and Weston NHS Foundation Trust and University of Bristol, Bristol, UK; 7 Population Health Research Institute, St George’s University London, London, UK; 8 The UK Teratology Information Service, Newcastle Upon Tyne Hospitals NHS Foundation Trust, Newcastle upon Tyne, UK; 9 Epidemiology and Population Health, London School of Hygiene and Tropical Medicine, London, UK

**Keywords:** drug utilisation, epidemiology, pregnancy, antidepressive agents, general practice

## Abstract

**Background:**

Antidepressant use is increasing during pregnancy but estimates of prevalence and patterns of prescribing are outdated.

**Aim:**

To describe the prevalence and patterns of antidepressant prescribing in and around pregnancy.

**Design and setting:**

This was a drug utilisation study using the UK’s Clinical Practice Research Datalink (CPRD) GOLD Pregnancy Register.

**Method:**

Using primary care prescription records, individuals were identified who had been prescribed antidepressants in and around pregnancy between 1996 and 2018 and the prevalence of prescribing during pregnancy over time was described. Those with ‘prevalent’ or ‘incident’ antidepressant use were defined, where the ‘prevalent’ group contained individuals who were prescribed antidepressants both before and during pregnancy, whereas individuals in the ‘incident’ group were newly prescribed antidepressants during pregnancy. Patterns of prescribing were then qualitatively compared between these two groups. The study also investigated post-pregnancy prescribing, as well as characteristics associated with antidepressant discontinuation anytime during pregnancy.

**Results:**

A total of 1 033 783 pregnancies were eligible: 79 144/1 033 783 (7.7%) individuals were prescribed antidepressants during pregnancy and 15 733/79 144 (19.9%) were in the ‘incident’ group. Antidepressant prescribing during pregnancy increased from 3.2% (556/17 653) in 1996 to 13.4% (3889/29 079) in 2018. Most women, both those whose antidepressants were ‘prevalent’ and ‘incident’ prescribed, discontinued their medication anytime during pregnancy (54.9% [34 801/63 411] and 59.9% [9427/15 733], respectively). Over half of those who discontinued during pregnancy were prescribed antidepressants in the 12 months after pregnancy (53.0%, 23 457/44 228). Younger age, previous stillbirth, and higher deprivation were associated with more frequent discontinuation anytime during pregnancy.

**Conclusion:**

Antidepressant prescribing during pregnancy has been increasing in the UK. Over half of the sample discontinued antidepressants at some point before the end of pregnancy, but post-pregnancy resumption of antidepressants was common. The results presented here highlight the benefit of counselling women when initiating antidepressants to support informed decision making.

## How this fits in

Antidepressant use during pregnancy is increasing but up-to-date estimates of prevalence and patterns of prescribing are unknown. It is important to maintain mental health, particularly during pregnancy, so the risk of destabilisation when discontinuing is important to consider among this population. The study showed that antidepressant prescribing during pregnancy has been increasing over time; discontinuation during pregnancy is common, as well as resumption in the 12 months after pregnancy. The results presented here highlight the benefit of counselling women of childbearing age on initiation of antidepressants to support informed decision making about their treatment if they were to become pregnant.

## Introduction

Antidepressants are widely prescribed medications^1,2^ and are used for a range of indications, predominantly depression and anxiety.^3,4^ Pregnancy is not a contraindication for antidepressants; however, the UK’s National Institute for Health and Care Excellence (NICE) lays out a series of recommendations for depression management before, during, and after pregnancy,^5–8^ mirrored by other guidelines updated in 2025.^9,10^ The guidance refers to regimen changes such as discontinuation (for less severe illness), dose tapering, and product switching if the risk of maternal condition destabilisation is lower than the potential risk to the fetus, assessed on an individual basis.^5,6^ ‘Risk to the fetus’ refers to both the uncertain effects of the medication *in utero* ^11–13^ and potential consequences of unmanaged maternal illness on the fetus via physiological imbalances or differences in demographic characteristics, such as smoking and poor diet*.* ^14–16^

Antidepressant use is increasing globally among women of a childbearing age outside of pregnancy.^17–20^ During pregnancy, previous data from the UK (excluding Wales) suggested that 3.7% of women who had a delivery (either live or stillbirth) between 2004 and 2010 were exposed to selective serotonin reuptake inhibitors (SSRIs) during pregnancy, dropping from 8.8% in the year before pregnancy.^21^ This drop may reflect the clinical guidance, where discontinuation has been recommended,^5^ or reflect stigmatisation of antidepressant use during pregnancy and limited evidence for their safety.^22^ Similar patterns of discontinuation have been found in a previous study of antidepressant use during pregnancy.^23^ As guidelines are updated based on emerging evidence, so do prescribing patterns, and it is important to monitor them for research and clinical purposes.

The Clinical Practice Research Datalink (CPRD) GOLD is a repository of UK primary care data.^24^ Previous studies on antidepressant utilisation during pregnancy have used the CPRD GOLD Mother–Baby Link, which includes all mother–baby pairs where both the mother and baby were registered with a CPRD GOLD practice.^21,23^ However, the current study used CPRD GOLD’s Pregnancy Register instead, capturing all pregnancy episodes in the CPRD GOLD population, regardless of delivery type or child registration with a CPRD GOLD practice.^25^ Trends in antidepressant prescribing during pregnancy between 1996 and 2018, patterns of antidepressant prescribing in and around pregnancy, and characteristics associated with discontinuation during pregnancy are described here.

## Method

### Data sources

CPRD is split into Aurum and GOLD. CPRD GOLD consists of primary care data from consenting general practices that use Vision software to capture patient data.^24^ It covers approximately 7% of the UK population and is broadly representative by age, sex, and ethnicity.^24^ CPRD GOLD contains information on prescriptions using British National Formulary (BNF) codes and diagnoses using Read codes. The CPRD GOLD Pregnancy Register contains algorithmically derived information on all pregnancy episodes in the CPRD GOLD population.^25^ CPRD GOLD is linked to external data sources, including Hospital Episode Statistics (HES, for ~75% of English practices^24^) using International Classification of Diseases Tenth Revision (ICD-10) codes, Office for National Statistics death registration data, and Index of Multiple Deprivation (IMD)^24^ patient- and practice-level quintiles (Supplementary Information S1).

All data in CPRD GOLD are pseudonymised which precludes the need for patient consent and details of CPRD’s safeguarding processes can be found online at the Medicines & Healthcare products Regulatory Agency website (https://www.cprd.com/protecting-patient-data and https://cprd.com/safeguarding-patient-data). Patient and public engagement was not undertaken as part of this study.

### Population

Using the CPRD GOLD Pregnancy Register, patients with an estimated pregnancy start date within an enrolment period of 1 January 1996 to 31 December 2018 that ended in either a loss or delivery were included. Eligibility included registration with an ‘up-to-standard’^24^ practice for ≥12 months before estimated pregnancy start (using last menstrual period, estimated due date, or imputed^25^) until the end of pregnancy, allowing sufficient time for collection of information before and during pregnancy. Each eligible pregnancy was followed up until the first of the following: transfer out of the practice, death, or the last collection date from the practice (the end of the study period, up to September 2021). The unit of measurement is ‘a pregnancy’; multiple pregnancies were included, considered once, and individuals who had >1 eligible pregnancy were included for each pregnancy. Unknown outcomes and conflicting pregnancies were resolved where possible as per Campbell and colleagues’ approach^26^ (Supplementary Information S2); unresolved pregnancies were dropped.

### Antidepressant prescribing

Antidepressants were defined using validated codelists and divided into SSRIs, serotonin-noradrenaline reuptake inhibitors (SNRIs), tricyclic antidepressants (TCAs), and ‘other’ antidepressants (Supplementary Table S1). Prescriptions filled in the 12 months before, during, and in the 12 months after pregnancy were identified.

Prescription length was calculated by dividing the quantity of tablets prescribed by the number of tablets to be taken daily to estimate the prescription end date. Hot-decking imputation^27^ was used where these data were missing. Daily dose in milligrams were calculated by multiplying the number of tablets prescribed per day by the number of milligrams delivered per dose. Daily dose in milligrams for each medication was then standardised to low, medium, or high based on dose distributions (Supplementary Information S3). Individuals with prescriptions of different products that overlapped by >4 weeks were defined as being on a ‘multidrug regimen’.

‘Antidepressant prescribed’ was defined as ≥1 prescription overlapping with the period of interest: the 12 months before pregnancy, during pregnancy (each week of gestation), and the 12 months post-pregnancy.

**Figure 1.**
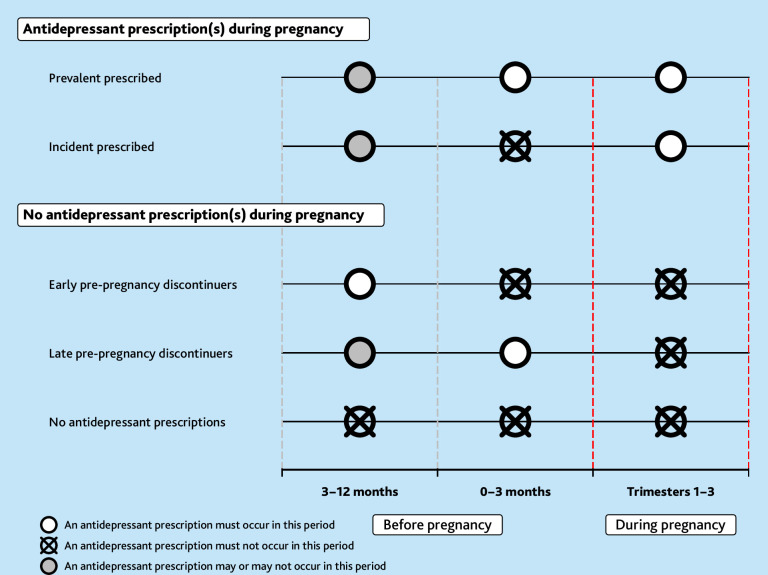
Prescription windows for pre- and during pregnancy.

Pre-pregnancy discontinuation was defined by prescription for antidepressants in the 12 months before pregnancy but not during pregnancy (Figure 1). Discontinuation during pregnancy was defined as antidepressant prescribing ending >2 weeks before the end of pregnancy. Discontinuation by trimester is described in Supplementary Information S4.

Among those who continued a single-drug regimen, antidepressant switching and dose changes were characterised; among those who continued a multidrug regimen, product adding, product dropping, and dose changes were characterised (Supplementary Figure S1, Supplementary Information S4). These patterns were explored among all individuals prescribed antidepressants, as well as those ‘prevalent’ and ‘incident’ prescribed (Figure 1).

Antidepressant prescribing after pregnancy was defined as those with ≥1 prescription in the 12 months post-pregnancy. Post-pregnancy prescribing was stratified by those newly prescribed post-pregnancy and those who were discontinuers from before and during pregnancy (follow-up sensitivity analysis described below).

### Possible indications

A list of licensed indications for antidepressants was compiled from the BNF and European Medicines Agency in the UK as of 2023. Then, corresponding Read and ICD-10 codes (lists verified by clinical authors) were identified that were applied to primary and secondary care data (where available) to identify the following: depression, anxiety, other mood disorders with a depressive element, eating disorders, pain, diabetic neuropathy, stress (urinary) incontinence, migraine prophylaxis, tension-type headache, and narcolepsy with cataplexy (Supplementary Information S5). Reporting indications for issued prescriptions is not a prerequisite of CPRD, so evidence of a possible indication may not denote the actual indication for which antidepressants were prescribed.

### Characteristics

Information on characteristics was abstracted for all eligible pregnancies. Demographics such as age (at the start of pregnancy), body mass index (BMI, around the start of pregnancy), ethnicity, socioeconomic position (proxied using practice-level IMD quintile),^28^ gravidity and parity (at the start of pregnancy), primary care consultations (in the 12 months before pregnancy), prescriptions of other medications (in the 12 months before pregnancy), and other diagnoses (ever before the start of pregnancy), were captured from relevant data sources and defined in Supplementary Table S2.

### Statistical analysis

The proportion of pregnancies in each year in individuals prescribed antidepressants during pregnancy and restricted to pregnancies ending in live births in sensitivity analysis were calculated. Antidepressant prescribing during pregnancy by UK region across the study period is shown.

Among eligible pregnancies, the proportion of individuals who were prescribed antidepressants before pregnancy was calculated and of these, the proportion who discontinued antidepressants before pregnancy was calculated.

Of those prescribed antidepressants during pregnancy, the study described discontinuation and continuation of a single- and multidrug regimen (that is, switching and dose changes), repeating analyses stratified by ‘prevalent’ and ‘incident’ prescribed. Trimester of discontinuation was explored by restricting data to those individuals with ≥29 completed weeks’ gestation. The primary patterns analysis was stratified by nulliparity, stringency of ‘incident’ definition (>12 months without an antidepressant prescription before pregnancy), delivery type, either deliveries or losses, and restricted to those with linked secondary care data.

Of those who were prescribed antidepressants after pregnancy, the proportion of individuals newly prescribed post-pregnancy and those resuming having discontinued before, or during, pregnancy was calculated. To explore ‘post-pregnancy’ compared with ‘postnatal’ prescribing, this analysis was stratified by delivery type in sensitivity analysis. The sample was also restricted to those with ≥12 months post-pregnancy follow-up.

Timing of depression and anxiety was characterised and the proportion of those prescribed antidepressants during pregnancy that had evidence of each possible antidepressant indication was calculated.

Logistic regression, minimally adjusted for pregnancy start year, was used to understand the relationship between characteristics and antidepressant discontinuation anytime during pregnancy. Each logistic regression model was a complete records analysis (CRA), so individuals were dropped in the event of missing data and the models were clustered by pregnant individual to account for those contributing >1 pregnancy to the analysis. A sensitivity analysis was run investigating the association between record missingness and discontinuation during pregnancy to assess the potential bias introduced in a CRA.^29^ All analyses were performed in Stata 17.0 and R 4.3.1.

## Results

### Study population

**Figure 2.**
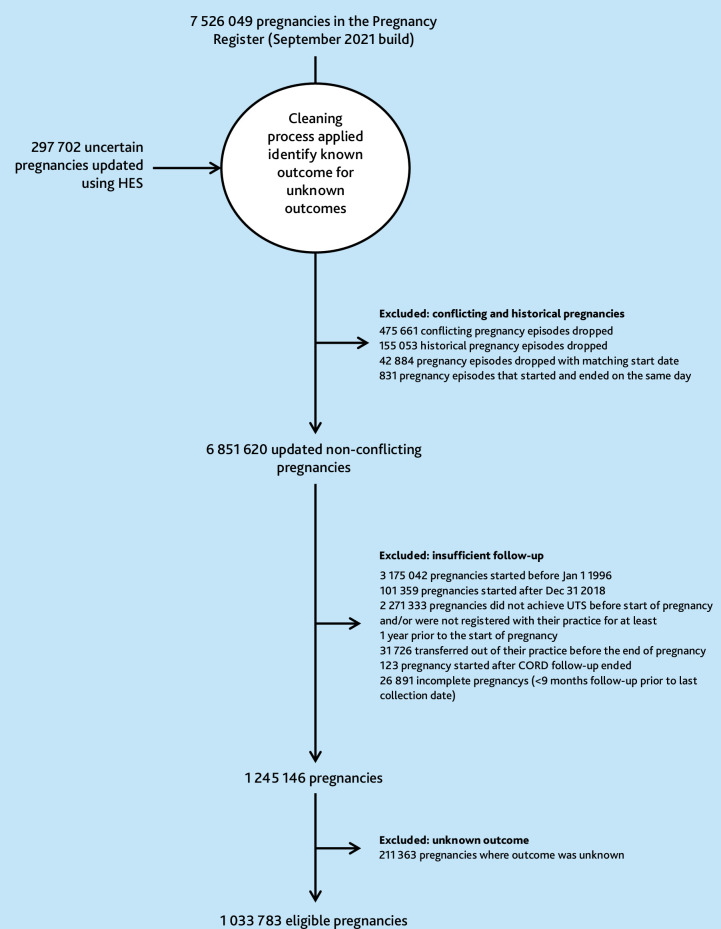
Detailed flow of pregnancies through the study. HES = Hospital Episode Statistics.

Of the pregnancies in the CPRD Pregnancy Register (September 2021), 1 033 783 were eligible (Figure 2). Pregnancies were excluded because they occurred outside the study window (3 276 401/7 526 049) or because of insufficient follow-up (2 271 333/7 526 049). Most pregnancies in the sample ended in a live birth (71.1%, 734 938/1 033 783), with 12.3% (127 521/1 033 783) ending in miscarriage and 13.5% (139 585/1 033 783) ending in termination, among other outcomes (Supplementary Table S3). Those with ≥1 pregnancy within which antidepressants were prescribed contributed more pregnancies to the analysis than those without any antidepressant prescriptions during pregnancy. Citalopram was the most commonly prescribed antidepressant during pregnancy (Supplementary Table S4).

Among eligible pregnancies, 79 144/1 033 783 (7.7%) were prescribed antidepressants anytime during pregnancy (Table 1). Those prescribed antidepressants during pregnancy were more likely to smoke and live in the most deprived IMD quintile than those who were not prescribed antidepressants. Other mental health-related prescriptions were more commonly prescribed to women who were prescribed antidepressants during pregnancy (such as mood stabilisers). High-dose folic acid and antiemetics were more widely prescribed during pregnancy to those also prescribed antidepressants than those who were not (Supplementary Table S5).

**Table 1.** Distribution of characteristics among those prescribed antidepressants during pregnancy (proportions of additional characteristics among those prescribed and not prescribed provided in Supplementary Table S5)

Characteristics	Prescribed antidepressants during pregnancy^a^ (*n*/*N*)	%
**Total**	79 144/1 033 783	7.7
**Pregnancy start year**		
1996–2000	5234/120 490	4.3
2001–2006	19 738/306 175	6.5
2007–2012	28 643/372 539	7.7
2013–2018	25 529/234 570	10.9
**Age at start of pregnancy, years**		
<18	1117/38 836	2.9
18–24	19 196/234 583	8.2
25–29	20 632/265 993	7.8
30–34	20 545/287 482	7.2
≥35	17 654/206 889	8.5
**Practice Index of Multiple Deprivation (IMD)**		
1st quintile (least deprived)	10 316/163 727	6.3
2nd quintile	11 811/167 765	7.0
3rd quintile	14 390/189 474	7.6
4th quintile	18 433/231 787	8.0
5th quintile (most deprived)	24 194/281 030	8.6
**Ethnicity**		
White	51 322/639 193	8.0
South Asian	959/31 837	3.0
Black	513/16 920	3.0
Other	357/11 235	3.2
Mixed	429/6657	6.4
Missing	25 564/327 941	7.8
**History of pregnancy loss at the start of pregnancy**		
Miscarriage	15 405/162 414	9.5
Stillbirth	722/6345	11.4
Termination	19 360/174 264	11.1
**Parity at the start of pregnancy**		
0	29 080/489 830	5.9
1	26 667/348 858	7.6
2	14 754/132 186	11.2
≥3	8324/58 465	14.2
**Number of GP visits in the 12 months before pregnancy**		
0	5177/116 218	4.5
1–3	5291/266 266	2.0
4–10	26 633/426 917	6.2
>10	42 043/224 382	18.7
**Mental health problems ever before the end of pregnancy**		
Depression	63 795/263 063	24.3
Anxiety	38 266/164 115	23.3
Schizophrenia	883/2405	36.7
Eating disorders^b^	4142/19 635	21.1

a A prescription made during or overlapping with pregnancy.

b Anorexia nervosa, bulimia, and other disordered eating codes (codelist in Supplementary Information S5).

### Trends over time

**Figure 3.**
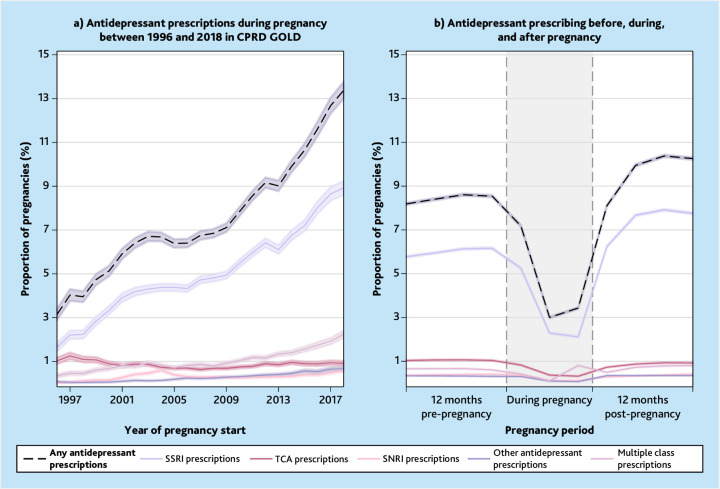
a) Antidepressant prescribing during pregnancy over time in Clinical Practice Research Datalink (CPRD) GOLD. b) Proportion of pregnancies in individuals who were prescribed antidepressants before, during, and after pregnancy. The denominator for trimesters two and three consists of those whose pregnancies reached trimesters two and three, respectively, that is, pregnancy losses in trimester one do not contribute to the denominator for trimesters two and three. SNRI = serotonin-noradrenaline reuptake inhibitor. SSRI = selective serotonin reuptake inhibitor. TCA = tricyclic antidepressant.

Antidepressant prescribing during pregnancy increased from 3.2% (556/17 653) in 1996 to 13.4% (3889/29 079) in 2018. Exclusive treatment with SSRIs has dominated antidepressant prescribing during pregnancy (Figure 3a). A similar increase was observed when restricting data to live births (333/12 752 [2.6%] in 1996 to 2582/20 492 [12.6%] in 2018) (Supplementary Figure S2) and when restricting data to pregnancies where ≥2 prescriptions were written during pregnancy (378/17 653 [2.1%] in 1996 to 3230/29 079 [11.1%] in 2018).

Wales had the highest overall proportion of antidepressant prescribing during pregnancy (9.5% of pregnancies, 12 185/128 681) and London had the lowest proportion (4.6% of pregnancies, 3606/77 744) (Supplementary Table S6).

### Patterns of prescribing

Of the 142 817 individuals prescribed antidepressants in the 12 months before pregnancy, 64.9% discontinued before the start of pregnancy (92 670/142 817).

Of the 79 144 pregnancies among individuals who were prescribed antidepressants during pregnancy (Figure 3b), 63 411/79 144 were in the ‘prevalent’ prescribed group (80.1% of antidepressant users during pregnancy). The remaining 15 733/79 144 (19.9%) were in the ‘incident’ prescribed group.

Most individuals in the ‘prevalent’ prescribed group discontinued antidepressants during pregnancy (54.9%, 34 801/63 411). Of the 42.8% (27 120/63 411) who continued a single-drug regimen throughout pregnancy, the majority appeared to continue their regimen with no changes (17 223/27 120, 63.5%), 22.9% (6209/27 120) changed their dose, and 8.6% (2329/27 120) switched to a different product. The remaining 5.0% (1359/27 120) had evidence of multiple regimen changes during pregnancy (Table 2).

**Table 2.** Patterns of prescribing during pregnancy

Pattern of prescribing during pregnancy	Total prescribed antidepressants during pregnancy	‘Prevalent’ prescribed group^a^	‘Incident’ prescribed group^b^
**All**	79 144 (100.0)	63 411 (100.0)	15 731 (100.0)
Discontinued during pregnancy^c^	44 228 (55.9)	34 801 (54.9)	9427 (59.9)
Continued^d^ a single-drug regimen throughout pregnancy	33 365 (42.2)	27 120 (42.8)	6245 (39.7)
Continued^d^ a multidrug^e^ regimen throughout pregnancy	1551 (2.0)	1490 (2.3)	~59 (0.4)^g^
**Continued a single-drug regimen throughout pregnancy**	33 365 (100.0)	27 120 (100.0)	6245 (100.0)
Antidepressant switched	2776 (8.3)	2329 (8.6)	447 (7.2)
Dose reduced	2237 (6.7)	2148 (7.9)	89 (1.4)
Dose increased	2236 (6.7)	1779 (6.6)	457 (7.3)
Dose fluctuated	2422 (7.3)	2282 (8.4)	140 (2.2)
More than one regimen change^f^	1473 (4.4)	1359 (5.0)	114 (1.8)
No changes to drug regimen	22 221 (66.6)	17 223 (63.5)	4998 (80.0)
**Continued a multidrug^e^ regimen throughout pregnancy**	1551 (100.0)	1490 (100.0)	~59 (100.0)^g^
Antidepressant added	225 (14.5)	215 (14.4)	10 (16.9)
Antidepressant dropped	207 (13.3)	193 (13.0)	14 (23.7)
Products added and dropped	294 (19.0)	281 (18.9)	13 (22.0)
Dose changes	83 (5.4)	83 (5.6)	<5
Multiple changes (to dose and product)	416 (26.8)	404 (27.1)	12 (20.3)
No changes to drug regimen	326 (21.0)	314 (21.1)	12 (20.3)

a Those who had ≥1 prescription for antidepressants in the 3 months before pregnancy and during pregnancy.

b Those who did not have a prescription for antidepressants in the 3 months before pregnancy but ≥1 prescription during pregnancy.

c Evidence of regimen changes before discontinuation *n *= 6998/44 228 (15.8%).

d Those who had an overlapping prescription with the end of pregnancy.

e Those prescribed ≥2 differing antidepressant products >5 days from the end of their current prescription.

f Those who experienced a switch in product as well as ≥1 dose change.

g The approximation is due to CPRDs suppression rule for patient counts <5.

Many individuals in the ‘incident’ prescribed group also discontinued during pregnancy (9427/15 733, 59.9%). Of the ‘incident’ prescribed group who continued a single-drug regimen throughout pregnancy (6245/15 733, 39.7%), the majority did not make any changes to their regimen (4998/6245, 80.0%). There was evidence of dose changes for a further 10.9% (686/6245), drug switching in 7.2% (447/6245), and multiple changes for 1.8% (114/6245) (Table 2).

When restricting the pattern analysis to specific time windows, a decrease in discontinuation (from 66.6% [3488/5234] 1996–2000 to 50.5% [12 892/25 529] 2013–2018) and a decrease in regimens with no changes (from 74.2% [1265/1706] to 63.0% [7482/11 878] over the same period) (Supplementary Table S7) was observed. A similar distribution of prescribing patterns was observed when using a more stringent definition of incident prescribing (Supplementary Table S8) and when stratifying by parity (Supplementary Table S9). When restricting to those who discontinued with ≥29 completed weeks’ gestation, the majority discontinued in trimester one: 81.8% (21 637/26 441) and 63.4% (4785/7547) in the ‘prevalent’ and ‘incident’ prescribed groups, respectively (Supplementary Table S10).

The primary analysis was restricted to deliveries, then to losses (Supplementary Table S3). The patterns of prescribing during pregnancy among deliveries was similar to the primary analysis, with 65.4% (34 234/52 306) discontinuing during pregnancy and 54.5% (9305/17 072) of those who were single-drug continuers making no changes to their regimen (Supplementary Table S11). Conversely, most women who experienced a loss continued antidepressants throughout pregnancy (62.8% [16 844/26 838]), reflecting the shorter length of gestation (Supplementary Table S12). When restricting to those with >1 prescription in pregnancy and then to those with linked HES data, the distribution of patterns did not change for either restriction (Supplementary Table S13 and Table S14).

In the 12 months after pregnancy, 15.8% (162 947/1 033 783) received ≥1 prescription for antidepressants (Supplementary Table S15), representing a slight increase from pre-pregnancy (Figure 3b). Of those who discontinued within 12 months before pregnancy, 34.2% (25 532/74 559) resumed antidepressant treatment in the 12 months after pregnancy. However, just over half of those who were ‘during pregnancy discontinuers’ resumed antidepressants in the 12 months after pregnancy (53.0%, 23 457/44 228) (Supplementary Table S15); when restricting data to first pregnancies 47.3% (5271/11 155) of those who discontinued during pregnancy, resumed in the 12 months after pregnancy (Supplementary Table S16).

By delivery type, 58.2% (5815/9994) of those who discontinued antidepressants during a pregnancy that ended in a loss then resumed post-pregnancy, compared with 51.5% (17 642/34 234) of those who discontinued during a pregnancy who had a delivery (Supplementary Table S17). When restricting data to those with ≥12 months of follow-up after the end of pregnancy, the proportions did not change (Supplementary Table S18).

### Possible indications

Of those prescribed antidepressants during pregnancy, 80.6% (63 795/79 144) had evidence of depression ever before the end of pregnancy (Table S5), of which only 4.9% (3866/79 144) was incident antenatal depression (Supplementary Table S19). Among the same group, 48.3% (38 266/79 144) had evidence of anxiety before the end of pregnancy, of which 3.0% (2388/79 144) was incident antenatal anxiety during pregnancy (Supplementary Table S19). Incident anxiety post-pregnancy was more common among those prescribed antidepressants during pregnancy, whereas incident post-pregnancy depression was more common among those not prescribed antidepressants (Table S19). Among those prescribed antidepressants during pregnancy, 9.6% (7578/79 144) had no evidence of an antidepressant indication (Supplementary Table S20).

### Characteristics associated with discontinuation

**Figure 4.**
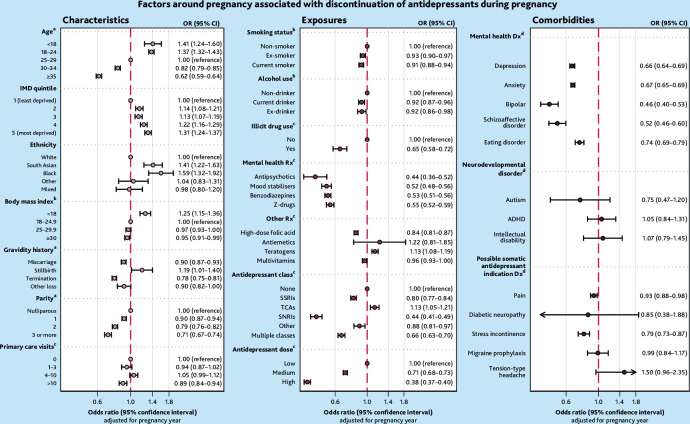
Maternal factors associated with discontinuation of antidepressants during pregnancy. ^a^At the start of pregnancy. ^b^Around the start of pregnancy. ^c^In the 12 months before pregnancy. ^d^Ever before the start of pregnancy. ADHD = attention deficit hyperactivity disorder. CI = confidence interval. Dx = diagnosis. IMD = Index of Multiple Deprivation. OR = odds ratio. Rx = prescription. SNRI = serotonin-noradrenaline reuptake inhibitor. SSRI = selective serotonin reuptake inhibitor. TCA = tricyclic antidepressant.

Younger age (<18 years: odds ratio [OR] 1.41, 95% confidence interval [CI] = 1.24 to 1.60) and 18–24 years: OR 1.37, 95% CI = 1.32 to 1.43), being underweight (OR 1.25, 95% CI = 1.15 to 1.36), and individuals who were more deprived (most deprived OR 1.31, 95% CI = 1.24 to 1.37) were more likely to discontinue antidepressants than comparators. Ethnicity was associated with discontinuation during pregnancy, where Black and South Asian individuals were more likely to discontinue than White individuals (Figure 4).

Discontinuing antidepressants during pregnancy increased the likelihood of having missing data for BMI, smoking, and alcohol use compared with continuing antidepressants throughout pregnancy (Supplementary Table S21).

## Discussion

### Summary

This study gives a detailed overview of antidepressant prescribing in and around pregnancy in the UK between 1996 and 2018, highlighting an increase from 3.2% (556/17 653) to 13.4% (3889/29 079) between 1996 and 2018. Utilising all prescriptions made during pregnancy, the study described patterns of antidepressant prescribing during pregnancy and showed the high resumption rate soon after the end of pregnancy among those who discontinued during pregnancy (53.0%, 23 457/44 228).

For both those in the ‘prevalent’ and ‘incident’ prescribed groups, the majority discontinued their regimen at some point during pregnancy (54.9% [34 801/63 411] and 59.9% [9427/15 733], respectively). In the ‘prevalent’ prescribed group who continued their single drug regimen throughout pregnancy, 63.5% (17 223/27 120) continued their regimen with no regimen changes, as opposed to 80.0% (4998/6245) of those in the ‘incident’ prescribed group. Most measured demographics were associated with discontinuation.

Those prescribed antidepressants during pregnancy have been shown to have greater needs and require more support during pregnancy.^30^ It is important to understand this group to provide the best care in and around pregnancy.

### Strengths and limitations

This study has several strengths. It is a large, population-based study that included pregnancies of all known outcomes, not restricted to live birth, using a validated pregnancy register linked to primary and secondary care.^25^ It is the first study, to the best of the authors’ knowledge, to discuss antidepressant prescribing alongside indication prevalence in and around the pregnancy period. Given that antidepressants are not sold over the counter in the UK and mostly prescribed in primary care, the authors are confident that the majority of antidepressant prescribing has been captured.

The study does, however, have several limitations. The number of pregnancies has been dropping in CPRD GOLD in the past decade, likely owing to more pregnant individuals self-referring to a midwife and circumventing the GP following policy changes in 2010.^31^ It is possible that women on antidepressants are more likely to report their pregnancy to the GP than women who are not, which would artificially inflate the proportion of women on antidepressants during pregnancy after 2010 in the eligible sample and make the increase in antidepressant prescribing during pregnancy look greater than is true in the general population. However, antidepressant prescribing during pregnancy increased from 3.2% (556/17 653) to 7.1% (4556/64 046) between 1996 and 2009 before changes in pregnancy reporting were enacted, reflecting trends outside of pregnancy among women of a childbearing age.

Despite updating unknown outcome pregnancies and conflicts where possible,^26^ 8.7% (651 014/7 526 049) of pregnancies in the sample were unresolved (in other words, there were no relevant data from HES for outcome identification) and were thus dropped. This may have introduced selection bias, and the authors of the current study may have incorrectly estimated the prevalence of certain prescribing practices or the associations between different demographics and discontinuation. Although gradual dose reduction is recommended when discontinuing antidepressants^32^ there was limited evidence of this in the prescription data. However, it is plausible that dose reductions may have been described by the prescriber in the free text that were then missed in the available structured data fields.

Pregnancy length was imputed for some pregnancies in the Pregnancy Register; this is more common for losses where less information on the pregnancy is available, potentially putting the study at risk of differential antidepressant exposure misclassification. The current study may have been more likely to misclassify losses as being in those who were prescribed antidepressants when they were not, thus may have overestimated antidepressant exposure and certain patterns among this group. Prescriptions of antidepressants was used to proxy exposure, but no information on dispensation or adherence was available, so some individuals may have been misclassified if they never filled or took their prescription. Identifying those on a multidrug regimen was challenging; it was difficult to differentiate an antidepressant switch from a multi-drug regimen in many cases and some multidrug regimens may have been misclassified as product switching.

Missing data was a problem in some of the covariates, such as smoking and BMI. In sensitivity analysis, an association between missingness in ethnicity, BMI, smoking, and alcohol use and an increased likelihood to discontinue antidepressants was observed, suggesting there may be a risk of bias in the CRA for these characteristics,^29^ so they should be interpreted with caution. Prescriptions made in hospital were not captured in these data, so pregnant women prescribed antidepressants solely in the hospital setting may have been misclassified as not prescribed antidepressants during pregnancy.

Individuals may have contributed >1 pregnancy to the analysis, which is not accounted for in the patterns analysis; however, the logistic regression models are clustered on the pregnant individual, and sensitivity analyses were included stratifying the patterns analysis on parity. Future studies may consider Poisson regression and other approaches to tackle this question.

### Comparison with existing literature

This study’s estimate of antidepressant prescribing during pregnancy is in line with the trajectory identified by Petersen *et al.* in 2011, who reported a fourfold increase in antidepressant prescribing during pregnancies that ended in a live birth between 1992 and 2006 in the UK.^33^ The upwards trend over time reflects the increased antidepressant prescribing in the general UK population over recent decades^19,34^ and shows the growing need for evidence-based advice on antidepressant use during pregnancy. Most individuals who were using antidepressants during pregnancy discontinued, predominantly in trimester one. NICE guidance notes that antidepressants can be used at any stage of pregnancy if clinically indicated but that their risks and benefits should be person centred.^5–7^ However, the evidence regarding risks and the efficacy of these guidelines in reducing them, is mixed or unknown.

In relation to patterns of prescribing, the findings were in line with previous literature.^21,23^ The study found continuation without dose changes was more common among the ‘incident’ than the ‘prevalent’ prescribed group, because of the likelihood that those in the ‘incident’ prescribed group would be initiated on and maintain a low dose if symptoms were managed.

It is important to note the high post-pregnancy antidepressant resumption rate among those who discontinued antidepressants during pregnancy, which remained high when stratified by delivery and pregnancy loss (51.5% [17 642/34 234] and 58.2% [5815/9994], respectively). A small study from France identified both benzodiazepine and anxiolytic use after pregnancy was higher than pre-pregnancy among those who discontinued antidepressants during pregnancy suggesting that symptoms may worsen when interrupting treatment.^35^ High antidepressant resumption rate may potentially reflect an exacerbation of illness during or after pregnancy.

Few studies have looked at characteristics associated with discontinuation of antidepressants during pregnancy. Prady *et al* reported similar percentages of discontinuation of medications for common mental disorders in different ethnic groups, but in a much smaller sample of 174 women who discontinued medication during pregnancy.^36^ Missing data and confounding should be factored into the interpretation of the analyses presented here but the findings are important nonetheless given the larger sample size and broad spectrum of characteristics explored.

### Implications for research and practice

The importance of descriptive epidemiology, here in the context of drug utilisation, cannot be underestimated.^37^ It underpins subsequent studies that aim to assess causality in an observational setting by highlighting important measured demographics among exposure groups of interest, key differences between them and potential comparator groups, and data pitfalls that might hinder causal inference. The current study provides a useful resource for both researchers hoping to contribute high-quality evidence regarding the safety of antidepressant use during pregnancy and clinicians who are interested in the trends in different prescribing patterns in and around pregnancy.

The results presented here highlight the benefit of counselling women of childbearing age on initiation of antidepressants that contextualises relative risk using absolute risks to support informed decision making if they were to become pregnant. Although from this study it is not known why people discontinued,^38^ transparency surrounding the way antidepressants are being prescribed in primary care in turn advocates for enhanced monitoring and the provision of non-pharmacological mental health treatments for women who discontinue. The findings promote discussions about the use of antidepressants during pregnancy, allowing for safer discontinuation strategies, such as gradual tapering, where appropriate. The high rates of postpartum resumption of antidepressants emphasise the importance of thorough mental health discussions at the 6-week postnatal check, particularly regarding the safety of antidepressant use during breastfeeding. The 2023 MBRRACE-UK report showed that suicide is still the leading cause of direct death in the 6 weeks to 12 months postpartum,^39^ reinforcing the importance of mental health care during this period.

The identification of an association between younger age, previous stillbirth, ethnicity, and higher deprivation with discontinuation during pregnancy highlighted vulnerable groups that may require additional support in primary care and suggests areas of continued research focus to better understand patient groups.

In conclusion, antidepressant use during pregnancy increased between 1996 and 2018 in the UK, from 3.2% (556/17 653) to 13.4% (3889/29 079). Over half of individuals prescribed antidepressants during pregnancy discontinued at some point before the end of pregnancy (55.9%, 44 228/79 144); the resumption rate in the 12 months after pregnancy was high (53.0%, 23 457/44 228) among these individuals. Future studies might leverage trajectory modelling to assess the impact of different antidepressant prescribing patterns on maternal health, primarily to address the dearth of evidence for antidepressant effectiveness during pregnancy.

## Data

All scripts and codelists are open source and can be accessed at: https://github.com/flozoemartin/Patterns/.

Data can only be accessed with ethical approval from CPRD.
